# Diet and gut microbiome interactions of relevance for symptoms in irritable bowel syndrome

**DOI:** 10.1186/s40168-021-01018-9

**Published:** 2021-03-26

**Authors:** Julien Tap, Stine Störsrud, Boris Le Nevé, Aurélie Cotillard, Nicolas Pons, Joël Doré, Lena Öhman, Hans Törnblom, Muriel Derrien, Magnus Simrén

**Affiliations:** 1 Danone Nutricia Research, Palaiseau, France; 2 Department of Internal Medicine and Clinical Nutrition, University of Gothenburg, Gothenburg, Sweden; 3 MGP MetaGénoPolis, INRA, Université Paris-Saclay, Jouy en Josas, France; 4 Department of Immunology and Microbiology, Institute of Biomedicine, University of Gothenburg, Gothenburg, Sweden; 5 Center for Functional Gastrointestinal and Motility Disorders, University of North Carolina, Chapel Hill, NC USA

**Keywords:** Diet, Microbiome, IBS, Function

## Abstract

**Background:**

While several studies have documented associations between dietary habits and microbiota composition and function in healthy individuals, no study explored these associations in patients with irritable bowel syndrome (IBS), and especially with symptoms.

**Methods:**

Here, we used a novel approach that combined data from a 4-day food diary, integrated into a food tree, together with gut microbiota (shotgun metagenomic) for individuals with IBS (*N* = 149) and healthy controls (*N* = 52). Paired microbiota and food-based trees allowed us to detect new associations between subspecies and diet. Combining co-inertia analysis and linear regression models, exhaled gas levels and symptom severity could be predicted from metagenomic and dietary data.

**Results:**

We showed that individuals with severe IBS are characterized by a higher intake of poorer-quality food items during their main meals. Our analysis suggested that covariations between gut microbiota at subspecies level and diet could be explained with IBS symptom severity, exhaled gas, glycan metabolism, and meat/plant ratio. We provided evidence that IBS severity is associated with altered gut microbiota hydrogen function in correlation with microbiota enzymes involved in animal carbohydrate metabolism.

**Conclusions:**

Our study provides an unprecedented resolution of diet-microbiota-symptom interactions and ultimately guides new interventional studies that aim to identify gut microbiome-based nutritional recommendations for the management of gastrointestinal symptoms.

**Trial registration:**

This trial was registered on the ClinicalTrials.gov, with the registration number NCT01252550, on 3rd December 2010.

Video abstract

**Supplementary Information:**

The online version contains supplementary material available at 10.1186/s40168-021-01018-9.

## Background

Irritable bowel syndrome (IBS) is one of the most common gastrointestinal disorders, affecting between 5 and 18% of the population, depending on geographical region [1, 2]. However, the effectiveness of treatment options for this common disorder is limited, and IBS is associated with profound reductions in health-related quality of life and huge societal costs [3]. Most individuals with IBS report the triggering or worsening of symptoms following food intake [4]. Therefore, the interest in the IBS symptoms’ dietary management has increased, particularly over the last decade. Recent clinical studies have shed light on the importance of specific food items in exacerbating gastrointestinal (GI) symptoms. However, the optimal dietary recommendations for individuals with IBS remains uncertain [4]. Diet, particularly long-term eating habits, is known to be one of the drivers of microbiota variation. Detailed tracking of covariations between the gut microbiota and diet demonstrated insight into the clearly personalized responses to variation in dietary intake [5, 6].

In population-based cohorts, significant associations have been found between dietary factors and interindividual distances in microbiota composition [7–11]. Culture-based studies have shown that strains from a given species may share metabolic activities but may also display differences [12, 13]. Consistent with this finding, recent metagenomics-based studies using strain-level profiling tools have revealed functional variation within species associated with dietary habits [14–16].

Carbohydrates are among the food components that can exacerbate symptoms, particularly those that are incompletely absorbed in the small intestine, which lead to gas accumulation, including hydrogen and methane, when microbial fermentation occurs in the colon [17]. These carbohydrates include, among others, short-chain fermentable carbohydrates (fermentable oligosaccharides, disaccharides, monosaccharides, and polyols (FODMAP) [18], and the metabolism of these may vary based on gut microbiota capacity to encode enzymes for metabolizing food glycans (CAZy or carbohydrate-active enzymes).

We previously showed that the gut microbiota is associated with symptom severity in IBS and identified a microbial signature for IBS severity that was not associated with the intake of macronutrients [19]. Although considered to be associated with symptom generation in individuals with IBS, details about how the combination of diet and gut microbiota affects symptoms remain unknown [19–21]. Therefore, in this study, we investigated the relation between digestive symptoms and extensive datasets of diet and the gut microbiota of individuals with IBS, with a focus on those with severe symptoms, further referred to as severe IBS, making use of a whole-metagenomics sequencing approach and categorized dietary intake based on a 4-day food diary.

First, using a diet index, we showed that individuals with severe IBS symptoms are characterized by a higher intake of food items of poorer quality during their main meals. Then, combining co-inertia analysis and linear regression analysis suggested that gut microbial hydrogen metabolism and dietary profile are associated with IBS symptom severity. Additionally, our study further suggests that specific hydrogenases were associated with gut microbiota function in terms of the CAZy involved in animal carbohydrate metabolism.

## Material and methods

### Subject recruitment and study design

Adult patients aged 18–65 years fulfilling the Rome III criteria for IBS [22] were prospectively included at a secondary/tertiary care outpatient clinic (Sahlgrenska University Hospital, Sweden). The diagnosis was based on a typical clinical presentation and additional investigations, if considered necessary by the gastroenterologist (HT or MS). Exclusion criteria included the use of probiotics or antibiotics during the study period or within the month preceding inclusion, another diagnosis that could explain the GI symptoms, severe psychiatric disease as the dominant clinical problem, other severe diseases, and a history of drug or alcohol abuse. Patients with severe heart disease, kidney disease, or neurological disease, as well as other GI diseases that could explain the symptoms, were not included. The healthy control group was recruited through advertisements and checked by interview and with a questionnaire to exclude chronic diseases and any current GI symptoms. The healthy controls completed a checklist consisting of the presence and severity of ten different GI symptoms (no, mild, moderate, severe, very severe) the previous week. To qualify as healthy control, no more than one symptom of maximum mild severity was allowed. The food diary was performed approximately 10–14 days before the stool sample collection. A bowel habit diary based on the Bristol Stool Form Scale was completed for 14 days before the stool collection to allow IBS subtyping according to the ROME III criteria [22]. Breath tests were done approximately 10–14 days after the stool sample collection. Concomitant medication was also recorded and previously reported [19].

All participants gave written informed consent for participation after receiving verbal and written information about the study. The Regional Ethical Review Board at the University of Gothenburg approved the study before the start of the inclusion period.

### Subject characterization

Demographic information and body mass index were obtained for all subjects. Individuals with IBS reported their current use of medications and completed questionnaires to characterize their symptom severity and bowel habits: the IBS Severity Scoring System (IBS-SSS) [23], a 4-day food diary, and a 2-week stool diary based on the Bristol stool form scale. IBS severity was assessed with validated cutoff scores for the IBS-SSS (mild IBS: IBS-SSS < 175, moderate IBS: IBS-SSS = 175–300, severe IBS: IBS-SSS > 300). Oral–anal transit time (radiopaque marker study) [24] and exhaled H_2_ and CH_4_ levels after an overnight fast (i.e., with no substrate intake preceding the test) were also determined for individuals with IBS. Exhaled CH_4_ and H_2_ levels were determined after an overnight fast (i.e., not after the intake of any substrate) and after the subjects had received thorough instructions to avoid a diet rich in fiber and poorly absorbed carbohydrates the day before the test. Exhaled H_2_ and CH_4_ levels were determined in parts per million in end-expiratory breath samples collected in a system used for the sampling and storage of alveolar air (GaSampler System; QuinTron Instrument Company, Milwaukee, WI) immediately analyzed in a gas chromatograph (QuinTron Breath Tracker; QuinTron Instrument Company).

### Dietary intake

All subjects completed a paper-based diet record, in which all foods and drinks consumed during four consecutive days (Wednesday–Saturday) were reported. Oral and written instructions were given to the patients on how to record their dietary intake, and patients were told to keep to their regular diet during the recording days. The type of food and the time at which it was consumed were noted, with quantification in grams, according to the use of household utensils (e.g., tablespoons) or the number of slices, for example. Cooking methods and the contents of food labels were noted where applicable. All diet records were entered into a special version of Dietist XP 3.1 software (Kostdata.se, Stockholm, Sweden), which calculates the energy and nutrient composition of foods. The software was linked to a Swedish Food Composition Database provided by the National Food Agency in Sweden (https://www.livsmedelsverket.se/) and to a Swedish database with FODMAP content, developed in-house [25]. This database contained information about fructose, fructan, lactose, galacto-oligosaccharide (GOS), and polyol content (g/100g) from published sources [26]. All diet records were entered into the software by a trained dietician. Excess fructose levels were calculated from data for fructose and total monosaccharide content from diet records. Glucose and fructose are the dominant monosaccharides in foods. If glucose content was higher than fructose, then the excess fructose variable was assigned a value of 0 (for each separate meal). For the reported intake of FODMAPs, outliers were defined as values exceeding the mean ± 4 SD.

Nutrient intakes were first summarized for each meal, and then per day, and were finally determined as the mean intake for all 4 days. A cutoff value was set for energy intake, and subjects reporting energy intake levels below 800 kcal/day or exceeding 4500 kcal/day were excluded to remove reports corresponding to an implausible habitual intake. No subjects exceeded these limits.

### FSA-NPS diet index

The FSA-NPS (British Food Standards Agency Nutrient Profiling System) [27] score was calculated for all foods and beverages, as follows: points (0–10) are allocated for the content per 100 g in total sugars (g), saturated fatty acids (g), sodium (mg), and energy (kJ) and can be balanced by opposite points (0–5) allocated for dietary fiber (g), proteins (g), and fruits/vegetables/legumes/nuts (percent). The grids for point attribution were as described by Deschasaux et al. [28]. The FSA-NPS score for each food/beverage is based on a unique discrete-continuous scale ranging theoretically from − 15 (most healthy) to + 40 (least healthy). Besides, each food item was assigned to one of five groups: from A (high quality) to E (low quality) for a FSA-NPS score below 1, 2, 5, 9, and 40 for A, B, C, D and E, respectively, for drinks, and below 0, 3, 10, 18 and 40 for A, B, C, D and E, respectively, for all other foods. The overall diet index, FSA-NPS DI, was calculated as the energy-weighted mean of the FSA-NPS scores of all foods and beverages consumed, as described by Deschasaux et al. [28].

### Hierarchical food tree and UniFrac analysis

We used the hierarchical format of the Swedish Food Composition Database to categorize foods into a hierarchical tree, the food tree. Food items, their associated nutrients, and their corresponding hierarchical levels were downloaded from the Swedish Food Composition Database (https://www.livsmedelsverket.se/). These hierarchical levels corresponded to levels 3 and 4 in the food tree. We then grouped level 2 into five large categories: animal-based, plant-based, alcohol, fats, and others. Level 1 is the root of the food tree. We then divided level 4 into 85 subcategories, constituting level 5 in the food tree, as follows:

1. For each level 4 category, we extracted the nutrient content for each food.
2. We fitted a Dirichlet multinomial mixture (DMM) model based on nutrient content for each level 4 category.
3. The number of Dirichlet components that resulted in the minimum Laplace approximation has been selected.
4. Each DMM component was used to assign food items to a subcategory in level 5 of the food tree.

The hierarchical structure of the food tree is shown in Supplementary Table S1. We used the food tree to calculate unweighted UniFrac metrics between food diaries.

For instance, items from the “cheese” food category (level 3 in the food tree) are classified as “animal product” (level 1) and “dairy product” (level 2), and have been classified into three food-nutrient subcategories (level 4): high-fat cheese, low-fat cheese, and whey-based cheese. All downstream analyses were performed at the food-nutrient level (corresponding to level 4 in Table S1).

### Fecal sample collection and DNA extraction

Fecal samples from 138 subjects were collected in RNA Later solution (Ambion, Courtaboeuf, France). Fecal DNA was extracted by mechanical lysis (Fastprep® FP120) (ThermoSavant, Illkirch, France) followed by phenol/chloroform-based extraction, as previously described [19]. A barcoded fragment library was prepared for each sample, and DNA sequencing data were generated with SOLiD 5500xl sequencers (Applied Biosystems, Life Technologies, Villebon-sur-Yvette, France), resulting in a mean of 38 (SD 14) million sequences of 35-base single-end reads. High-quality reads were generated, with a quality score cutoff > 20. Reads with a positive match to human, plant, cow, or SOLiD adaptor sequences were removed. Filtered high-quality reads were mapped onto the MetaHIT 3.9 million gene catalog with METEOR software. The read alignments were performed in color space with Bowtie software (version 1.1.0), with a maximum of three mismatches and a selection of the best hit. Uniquely mapped reads (reads mapping to a single gene from the catalog) were attributed to the corresponding genes and used to construct a raw gene count matrix. If multiple alignments were found, counts were divided equally between the aligned genes.

### Metagenomics species pangenome analysis

Metagenomics species pangenomes (MSPs) are co-abundant gene groups that can be considered part of complete microbial species pangenomes. MSP gene content was extracted from a previous publication by Plaza-Onate et al. [29]. MSP gene content was subdivided into core and accessory genes. Gene annotations (KEGG orthology and CAZy family) were extracted from the paper by Li et al. [30]. Thus, MSP relative abundance was calculated for each sample, based on the median core gene abundance. Samples were attributed to an MSP subspecies based on accessory gene clustering, as follows:

1. Median read coverage and the 2.5 and 97.5% quantiles were calculated for each sample and MSP. An MSP was considered to be detected in the sample if it had a median coverage of more than 2.
2. Each accessory gene within the MSP with a read coverage between the 2.5 and 97.5% quantiles was considered to be detected. Below the 2.5% quantile, genes were considered to be absent, and above the 97.5% quantile, genes were considered to be present in multiple copies or to be conserved genes that might bias the estimation of coverage. A presence/absence binary gene matrix was therefore obtained for each MSP.
3. A Jaccard index between samples was calculated from the MSP binary matrix
4. Clustering was performed, with a partition around medoids over 100 bootstraps achieved with the *clusterboot* function of the *fpc* package (bootstrap method option *“subset”*). The number of clusters (i.e., MSP subspecies) was estimated based on mean silhouette width.

### Gut microbiome hydrogenase and CAZy analysis

Hydrogenase amino acid sequences were extracted from a previous study [31] and aligned, with BlastX software (version 2.7.1+), with 3.9 million gene catalogs. Best hits with an identity of more than 60% over a stretch of more than 40 amino acids were considered for downstream analysis.

### Statistical analysis

All statistical analyses were performed with R software (version 3.4.1). UniFrac distances between food diaries were calculated with the phyloseq R package, using the food tree as input. Jensen Shannon divergence (JSD) between metagenomes aggregated at MSP subspecies level was calculated according to a tutorial published by Arumugam et al. [32] implemented into BiotypeR R package (available on github tapj/BiotypeR). PERMANOVA analysis was performed with the vegan R package (Adonis, version 2.5). Principal coordinate analysis (PCoA), co-inertia analysis, and linear discriminant analysis were performed with the ade4 R package (version 1.5). To note, PCoAs on microbiota and diet distances were computed using all available samples, while co-inertia analysis was computed on common sub-samples from PCoAs components. Spearman correlation analysis was used to project features onto PCoA axes. Principal component linear regression analysis was used to train models to predict clinical, microbiome, and diet features (e.g., exhaled gas metabolism, symptom severity, meat/plant ratio) with the co-inertia axes. Spearman’s correlation analysis was performed on relative abundance data for genes aggregated at the CAZy family and hydrogenase levels. A network was generated for correlations with an absolute rho value above 0.4. Data were visualized with cowplot, ggraph, and ggplot2. *p* values were adjusted for multiple testing by Benjamini–Hochberg false-discovery rate correction when specified. Otherwise, *p* values are given at a 5% nominal level.

## Results

### Dietary habits of the study population using a food item-based tree

We investigated the association of GI symptom severity and patterns with diet and the gut metagenome in 52 healthy controls and 149 individuals with IBS (Table 1). The IBS symptoms were severe, according to the IBS Severity Scoring System (IBS-SSS) [23], in 65 of the 149 individuals with IBS. A 4-day food diary was obtained from 142 subjects (26 healthy controls and 49 individuals with severe IBS). Dietary macronutrients and micronutrients identified as consumed differentially between individuals with IBS and healthy controls or between patients with severe IBS symptoms and other individuals (healthy or non-severe, i.e., mild/moderate IBS symptoms) did not remain significant after correction for multiple testing (Fig. S1 and S2). This suggests that dietary data, aggregated at the micro- and macronutrients levels, differ neither between individuals with IBS and healthy controls nor between IBS patients with different levels of symptom severity.

**Table 1:** Characteristics of the study cohort

	Healthy controls	Individuals with IBS
N	52	149
Female	32 (62%)	105 (70%)
Age (year)	28 [26–37]	31 [25–43]
BMI (kg/m^2^)	22.4 [20.65–24.50]	22.39 [20.64–24.95]
Mild IBS symptoms (IBS-SSS)	N/A	24 (16%)
Moderate IBS symptoms (IBS-SSS)	N/A	50 (34%)
Severe IBS symptoms (IBS-SSS)	N/A	65 (44%)
Gut metagenomic profile only	26 (50%)	33 (22%)
Dietary profile only	16 (31%)	47 (32%)
Gut metagenomic and dietary profiles	10 (19%)	69 (46%)

*BMI*, body mass index; *IBS-SSS*, IBS Severity Scoring System; *N/A*, not applicable; dasta are shown as *n* (percentage) or median [interquartile range]. IBS-SSS was not available for 10 IBS patients

**Fig. 1:**
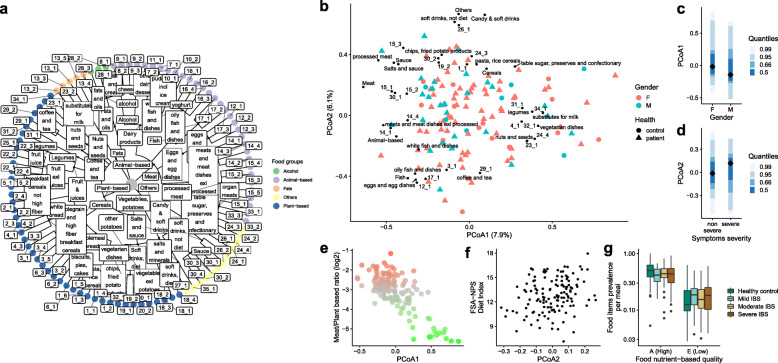
Quantity and quality assessment for dietary profiles and analyses of associations with gastrointestinal symptom severity. **a** Food item-based hierarchical tree based on the National Swedish food database and nutrient-based clustering. **b** Principal coordinate analysis of unweighted UniFrac distance between the dietary profiles of individuals. Food levels were projected onto the two first coordinates (PCoA1 and PCoA2) based on Spearman’s correlation analyses (see Supp Table 1 for the terminology of food level 4). Colors indicate the sex of the individual and the shape of the point indicates health status. **c** PCoA1 as a function of sex (*p* < 0.05). **d** PCoA2 as a function of IBS symptom severity (*p* = 0.06). **e** Log2 meat/plant ratio as a function of PCoA1 (rho = − 0.62, *p* < 0.05). The color gradient extends from red (all meat) to green (all plant-based foods). **f** FSA-NPS diet index as a function of PCoA2 (rho = 0.24, *p* < 0.05). **g** Prevalence of food items per meal as a function of FSA-NPS food quality and health status. Class A corresponds to high-quality food, whereas class E corresponds to low-quality food (*p* < 0.05)

Next, we assembled a food item tree, similar to that used by Johnson et al. [5], clustering food items based on nutritional content rather than the food item itself (see Material and methods section, “Hierarchical food tree and UniFrac analysis”). Participants consumed 966 different food items in total, which were aggregated into three hierarchical levels, based on the National Swedish food database, to build a hierarchical tree. A fourth level based on nutrient composition was also added, resulting in 85 food-nutrient groups (Fig. 1a and Table S1). The unweighted UniFrac distance between individuals was calculated using the four hierarchical levels of dietary data and subjected to principal coordinate analysis (PCoA) (Fig. 1b). Based on Spearman’s correlation coefficient, the four hierarchical food levels, including food-nutrient groups, could be projected onto principal coordinate axes 1 and 2 (PCoA1 and PCoA2). PCoA1 (7.9% of total variance) was associated with separating individuals according to their consumption of meat-based and plant-based food items (Fig. 1b). Further, PCoA2 (6.1% of total variance) separated individuals based on their consumption of unprocessed food items, such as fish and eggs, and processed food items, such as candy and fried potato products. Following the integration of selected clinical variables into the analysis, PCoA1, which was driven by meat-based and plant-based food items, was associated with sex (Fig. 1c, Mann–Whitney test, *p* < 0.05). PCoA2 tended to be associated with IBS severity, indicating that unprocessed and processed foods were associated with IBS symptom severity (Fig. 1d, Mann-Whitney test, *p* = 0.06). A meat-to-plant ratio was calculated for each subject, based on the aggregation of food items into these two categories (Table S2). PCoA1 was significantly associated with the meat/plant ratio (Fig. 1e, rho = − 0.62, *p* < 0.05), suggesting that the proportion of meat-based food relative to plant-based food was the major driver of dietary variation between subjects in our cohort.

According to the British Food Standards Agency Nutrient Profiling System (FSA-NSP), we then assessed diet quality by performing nutrient profiling for each food item and calculating an individual FSA-NPS Dietary Index (DI) score. A higher FSA-NPS DI reflects a lower nutritional quality of the foods consumed by the individual (“less healthy”). The median FSA-NPS DI was 13.75 (IQR 12.06–14.96) for healthy controls and 12.53 (IQR 11.33–13.91) for individuals with IBS (Mann–Whitney test, *p* > 0.05). While food tree PCoA2 tended to be associated with IBS symptom severity (see above), food tree PCoA2 was also associated with a lower overall dietary quality (Fig. 1f rho = 0.24, *p* < 0.05), indicating an association between IBS symptom severity and food quality (i.e., less “healthy food” in patients with more severe IBS symptoms). This led us to examine a potential direct link between IBS symptom severity and food quality during meals. With the FSA-NPS, food items could be classified into five groups (from high-quality A to low-quality E, see Methods). Individuals with severe IBS symptoms consumed a higher amount of lower-quality food items during their main meals than healthy controls and individuals with milder IBS symptoms (Fig. 1g, Mann–Whitney, *p* < 0.05 for high and low food qualities). The median ratio between high-quality and low-quality foods during the main meals was 5.8 for healthy controls and 2.7 for individuals with severe IBS, while there was no difference when snacks were included in the analysis (2.5- and 2.0-fold for healthy controls and individuals with severe IBS, respectively).

Finally, based on the PCoA results, we investigated whether FODMAPs (fermentable oligo-, di-, and monosaccharides and polyols) were associated with diet quality based on the hierarchical food tree. GOS and fructan intakes were positively associated with PCoA1 (rho = 0.28 and rho = 0.33 respectively, *p* < 0.05), suggesting that subjects who consumed high levels of plant-based food items had a diet enriched in GOS and fructans. Polyol intake was associated with PCoA2 (rho = − 0.31, *p* < 0.05), indicating that subjects who consumed high levels of processed foods and lower-quality food items had a diet enriched in polyols. The intake of lactose and fructose was not associated with PCoA1 or PCoA2, suggesting that their consumption was not a major driver of the overall variation in dietary intake or symptom generation in our study.

### Dietary profile associations with gut microbiota composition and function

As diet is a major driver of microbiota composition and function, we investigated whether differences, previously observed in dietary profile between study individuals, were associated with gut microbiota composition and functions. We have previously shown that enterotypes, assessed by 16S rRNA gene sequencing, could be separated into three microbiota communities based on Dirichlet multinomial mixture (DMM) modeling [19]. Enterotypes were not associated with dietary distance (PERMANOVA, *p* > 0.05), nor with meat/plant ratio (*p* > 0.05) or diet quality (FSA-NPS DI, *p* > 0.05), suggesting that the variation of diet within this cohort was not associated with that of global microbiota structures (see Supplementary Tables S2 and S3).

**Fig. 2:**
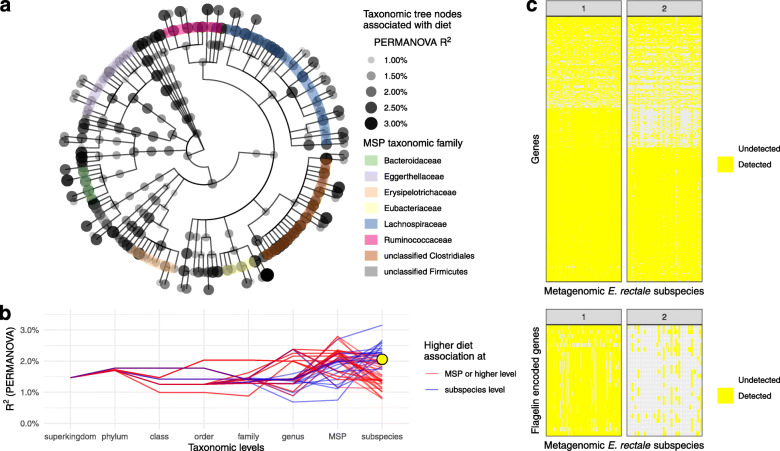
Dietary profile associations with gut microbial lineage and functions. **a** taxonomic tree of the microbial lineage with nodes corresponding to effect sizes of more than 2% (R2 assessed by PERMANOVA). The color code indicates the taxonomic family of the MSP taxonomic family. **b** Effect size of dietary variation (R2 assessed by PERMANOVA) as a function of taxonomic level. Red lines correspond to lineages for which effect size was greater at subspecies level. Yellow dot accounts for *Eubacterium rectale* subspecies 1. **c** Presence/absence heatmap showing the genes detected within each *Eubacterium rectale* subspecies with a specific focus on flagellin-encoded genes. Genes are represented as rows and samples as columns

Next, whole-metagenomic sequencing was performed in 138 individuals (102 individuals with IBS and 36 healthy controls), and among these subjects, both diet and metagenomics profiling were available in 79 subjects (Table 1). Metagenomic reads (with an average of 14 million reads per sample) were mapped onto a catalog of Metagenomic Species Pangenomes (MSPs) [29], yielding a total of 1661 MSPs. Based on per-individual genetic content, 166 of them were further divided into 523 subspecies, corresponding to a mean of 75.3% of the metagenome read mass. The remaining 1495 MSPs were not assigned to MSP subspecies (MSP_unassigned). We then used the taxonomic tree to investigate the effect on each node’s dietary profile variation, from phylum to subspecies level (PERMANOVA test). Each microbial lineage with at least one node with an effect size of more than 2% was selected with no filter applied on statistical significance (Fig. 2a). Depending on taxonomic lineage, dietary variation was either better explained at the species (i.e., MSPs) or subspecies level (Fig. 2b). For example, the MSP assigned to *Eubacterium rectale* was less associated with diet than its two subspecies. In particular, the relative abundance of the *E. rectale* subspecies with flagellin-encoding genes (Fig. 2c) correlated with a diet enriched in meat-based products (rho = 0.23, *p* = 0.05) and with vitamin B12 intake (rho = 0.31, *p* < 0.05).

### Association of microbiota function and dietary variation with clinical parameters and gas metabolism

**Fig. 3:**
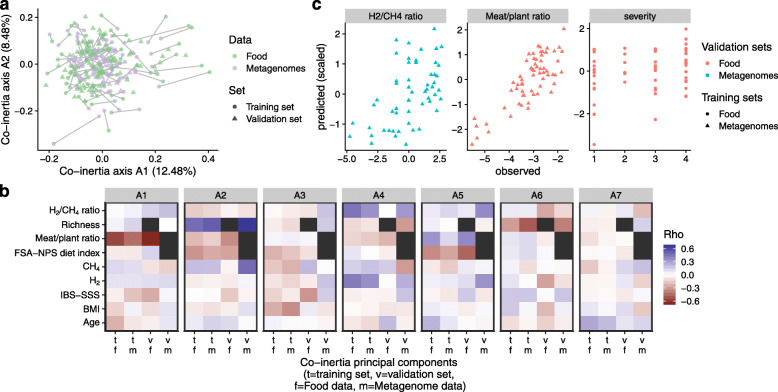
Co-inertia analysis associates microbiota profiles with dietary profiles, together with gas metabolism and symptom severity. **a** Co-inertia scatter plot with the training set (*n* = 79) including both metagenomic and dietary data, and the test set (*n* = 122) for which only dietary or metagenomics data were available. Individual coordinates for the test set were computed from their PCoA coordinates with the co-inertia model. Colors indicate the data source (diet or metagenomic). **b** Heatmap of Spearman’s correlations between the first seven co-inertia axes and clinical, gas metabolism, gene richness and dietary data. The color indicates the strength of the correlation. Black squares indicate missing data. *v,* validation set; *t*, training set; *m*, microbiota; *f*, food. **c** Scatter plots of the relation between predicted (linear regression) and observed data for H_2_/CH_4_ ratio (*r* = 0.53, *p* < 0.05), meat/plant ratio (*r* = 0.69, *p* < 0.05) and symptom severity (*r* = 0.28, *p* < 0.05) (based on the first 5 co-inertia components). The color indicates the validation set source, whereas the shape of the points indicates the training set source (dietary or metagenomic). IBS symptom severity groups (healthy, mild, moderate, and severe) were coded from 1 to 4, respectively

As dietary variation was better explained at the species (i.e., MSPs) or subspecies level MSP, we further combined microbiota profile variation (JSD distance at subspecies and MSP_unassigned) with dietary profile variation. Using a co-inertia approach on PCoA components, we explored the complex association between gut microbiota, diet, and multiple explanatory variables in 79 subjects for whom both dietary and microbiota datasets were available (training set, co-inertia RV coefficient = 0.59). Individuals with only one of these datasets (microbiota or diet, Table 1) constituted the test set (*N* = 122). All 201 study subjects (training and test sets) were projected onto the same hyperspace based on their gut microbiota and dietary profiles (Fig. 3a). We then investigated how this common projection of gut microbiota and diet was associated with clinical parameters. The relation of microbiota and dietary profiles to gas metabolism and clinical parameters was investigated by extracting the first co-inertia coordinates from both the training and test sets. Then, we correlated them with variables, including BMI, age, IBS symptoms severity score, exhaled gas metabolism (H_2_ and CH_4_), microbiota gene richness, and dietary variables (meat/plant ratio, diet quality) (Fig. 3b). The first seven coordinates (axis A1 to axis A7, Fig. 3c), accounting for 50% of covariation, were retained. Consistently for both the training and test sets, the meat/plant ratio was associated with axis A1, whereas microbial gene richness, diet quality, and exhaled CH_4_ were associated with axis A2 (Table S3). This suggests that the meat/plant ratio was the main tested factor explaining microbiota and dietary covariations independently of gene richness, diet quality, and exhaled CH_4_. Although weaker, consistent correlation directions for axis A1, in both the test and training set, were detected for exhaled H_2_ and IBS symptom severity. Since CH_4_ metabolism is dependent on H_2_ metabolism, the ratio of exhaled H_2_ to exhaled CH_4_ for each subject was calculated. This ratio was consistently correlated with axis 4 in both the food and microbiota in the training sets and microbiota in the test set, suggesting that H_2_/CH_4_ metabolism can be explained by metagenomic and dietary profile variations.

For the prediction of each clinical variable, we used a machine learning approach by constructing regression models from the first five co-inertia coordinates (40% of the variance) extracted from the microbiota and dietary datasets. Co-inertia models trained on microbiota and validated on dietary data were assessed for robustness by regression analysis (Fig. 3c). For example, using a model fitted on the microbiota training set, H_2_/CH_4_ ratio could be predicted from the microbiota test set (Pearson *r* = 0.53, *p* < 0.05). Similarly, using a model fitted on the microbiota training set, the meat/plant ratio could be predicted from the dietary test set (*r* = 0.69, *p* < 0.05). Using another model fitted on the dietary training set, IBS symptom severity could be predicted from the dietary test set (*r* = 0.28, *p* < 0.05).

### Carbohydrate-associated enzymes and hydrogenases encoded by metagenomes are associated with IBS symptom severity

As carbohydrates belong to the food items that can exacerbate symptoms related to gas accumulation, we further explored the relationship between CAZy and hydrogenases. Metagenomes were clustered according to DMM models based on the relative abundance of CAZy. Using the minimum Laplace approximation, study subjects could be divided into three distinct CAZy clusters (CAZotype) that were significantly associated with enterotypes (chi^2^, *p* < 0.05, Fig. S4), suggesting a link between carbohydrate metabolism and enterotypes. In contrast to enterotypes, CAZotypes were associated with the diet profile variation (PERMANOVA test, *p* < 0.01).

**Fig. 4:**
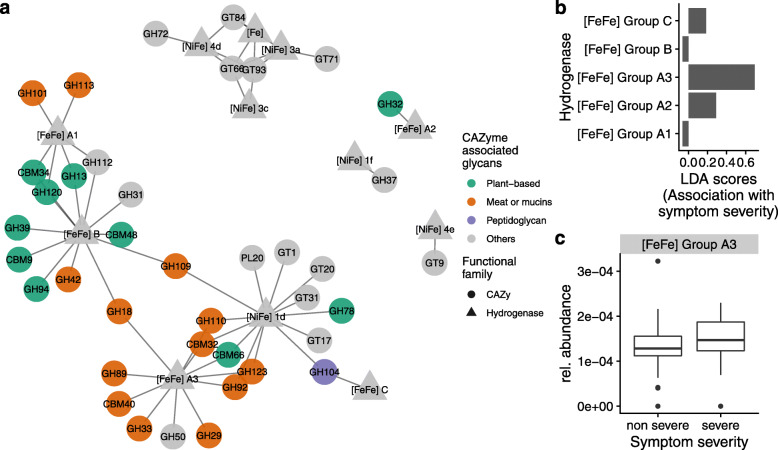
Association between hydrogen levels, glycan metabolism and GI symptom severity. **a** Network of associations between CAZy family and hydrogenase group. An edge represents absolute Spearman’s correlation coefficients above 0.4. All kept associations were positive. The node shape indicates CAZy family (circle) and hydrogenase (triangle). The color code indicates the known glycan substrates of the CAZy family. **b** Linear discriminant analysis (LDA) scores for prediction of IBS severe vs control as a function of gut metagenomic hydrogenase [FeFe] group. **c** Relative abundance of gut metagenome hydrogenase [FeFe] A3 as a function of health status

We further investigated potential links between CAZy and hydrogenases, encoded by gut metagenomes, and symptom severity. CAZy were subdivided into broad substrate categories (plant cell wall carbohydrates, animal carbohydrates, peptidoglycan, and others (starch/glycogen, sucrose/fructans, fungal carbohydrates, and dextran) [33], whereas hydrogenases were classified according to their active metal site [31]. A network based on Spearman’s correlation between the CAZy family and hydrogenases was constructed (Fig. 4a). Network analysis showed that a specific hydrogenase involved in hydrogenotrophy, [FeFe] group A3, was associated with eight different CAZy families involved in the metabolism of animal carbohydrates (mucin- or meat-derived) (Fig. 4a). One plant-based CAZy family, a carbohydrate-binding module known to target the terminal fructoside residue of fructans, was also associated with [FeFe] A3 hydrogenase. Hydrogenase from the [FeFe] group B was associated with seven plant-based CAZy families, including enzymes involved in starch and xylose metabolism. This suggests that the abundance of [FeFe] hydrogenase in gut metagenomes is associated with the metabolism of dietary and host glycans. Finally, we assessed the relation between the relative abundance of [FeFe] hydrogenases and IBS symptom severity. A linear discriminant analysis showed that [FeFe] A3 hydrogenase was a strong predictor of IBS symptom severity (Fig. 4b). Indeed, [FeFe] A3 hydrogenase presented higher relative abundance in individuals with severe IBS compared with healthy controls (Fig. 4c). Overall, these findings indicate a link between carbohydrate metabolism and symptom generation in IBS.

## Discussion

In this study, we have demonstrated that individuals with severe IBS are characterized by a higher intake of food items with lower overall quality during their main meals. Our study further provides evidence that IBS severity is associated with altered gut microbiota hydrogen function in correlation with CAZy involved in animal carbohydrate metabolism. A combination of co-inertia analysis and linear regression analysis also suggested that covariations between gut microbiota and diet were associated with IBS symptom severity, exhaled gas, glycan metabolism, and meat/plant ratio.

Dietary components are often analyzed separately without taking nutritional similarity or relatedness into consideration. In our study, we could not identify an association between macronutrient [19] and gut microbiota composition. However, the recently developed approach to structure food items into a hierarchical tree [5, 15], similar to that used for microbiota phylogenetic tree analysis, may facilitate the detection of associations that are otherwise difficult to identify, e.g., for nutrients that are not well captured, particularly those known to modulate the microbiota, including fiber and polyphenols [5, 34]. We took advantage of this approach to decipher diet–gut microbiota interactions in the context of IBS. We modified it, using nutrient intake derived from a 4-day food diary rather than food items themselves, making it possible to separate closely related food items with differences in only a few nutrients. Based on the food item tree analysis, the meat/plant ratio was the variable that most strongly discriminated against among subjects within the study cohort. Furthermore, we have also shown that the overall quality of dietary intake based on FSA-NSP profiling did not differ significantly among IBS subjects or between individuals with severe IBS and healthy controls [28].

However, individuals with severe IBS consumed a higher proportion of food items that can be considered as low quality (“less healthy”) as part of their main meals. This suggests that the quality of food in the main meals, and not only the overall diet, is relevant when studying symptoms. Given the intricate relation between dietary habits, potential self-restriction, and symptoms in IBS, it is difficult to disentangle these factors. However, as our cohort was naïve to current IBS dietary recommendations such as low FODMAP diet, self-restriction is not a plausible explanation for these associations. For example, in our study, galactooligosaccharides and fructan intakes were not associated with reports of more severe symptoms in IBS.

Based on the current finding that the individuals with severe IBS symptoms differed from the other subjects in terms of dietary habits, together with our previous identification of a specific gut microbial signature associated with severe IBS symptoms [19], we further explored the association between dietary habits and gut microbiota composition and function. In this study, we did not find a direct relation between diet profiles and variation in the gut microbiota composition, using 16S rRNA gene analysis. This might be due to a combination of low sample size and low variability in dietary habits (intra-population), in contrast to other studies that included larger populations [7].

Using a new approach combining shotgun metagenomic microbiota and food trees, we identified the most associated taxa associated with the dietary variation. Analyses of the gut microbiota beyond the species level revealed associations that were not detected in analyses at the species level, and for example, *Eubacterium rectale* subspecies harboring flagellin-encoding genes were associated with a predominantly meat-based diet. This complements a previous study that reported that two of the three *E. rectale* subspecies consistently harbored an accessory pro-inflammatory flagellum operon associated with lower gut microbiota community diversity, higher host BMI, and higher fasting blood insulin levels [35]. Future studies with a larger sample size would enable a better characterization of the association between different subspecies of *E. rectale* and the dietary profile [36, 37].

We further explored the possible relationship between functional variation in gut microbiota, diet, and symptoms. So far, most IBS studies focused on symptoms associated with specific dietary ingredients and independently of gut microbiota. Using a combination of principal component analysis and linear regression, we could predict diet’s main variation factor (meat/plant ratio) based on the gut microbiota profiles of a given individual. Several studies did not observe differences between animal- and plant-based foods in relation to gut microbiota composition based on adherence to omnivorous, vegan, or vegetarian diets (reviewed in [38]). This is potentially due to the lack of precision of dietary pattern adherence and/or to gut microbiota resolution [39]. In this study, we were able to accurately infer the meat/plant ratio from the food diaries. Additionally, this approach provides elements to support the hypothesis that the dietary profiles and the gut microbiota are associated with IBS symptom severity and exhaled gas, resulting from microbial fermentation of dietary ingredients.

As carbohydrates are among the food items that may trigger gas accumulation and symptoms, we further explored the relation between CAZy and hydrogenases. In this study, the abundance of hydrogenases associated with IBS symptom severity was positively correlated with a higher abundance of CAZy involved in the metabolism of dietary and host animal glycans. In addition to carbohydrate metabolism, gases, including hydrogen, are of considerable interest in the context of gut disorders [40]. Hydrogen (H_2_) is formed in large volumes in the colon as an end-product of carbohydrate fermentation [41–43]. Recent metagenomics studies have identified three types of hydrogenases involved in H_2_ metabolism [31, 44]. Interestingly, we found that individuals with severe IBS symptoms had a higher abundance of a specific type of hydrogenases involved in the metabolism of H_2_. This is in agreement with our previous finding that individuals with IBS with high exhaled H_2_ levels had a distinctive ratio of active gut microbiota members independent of gastrointestinal symptom pattern or severity [45]. In this study, we confirmed the important role of hydrogen metabolism and provided novel insight regarding the identification of specific hydrogenases of relevance for symptom generation in IBS. We suggest that analysis of hydrogenases should be included in future IBS and overall diet–microbiota studies.

This study has some limitations. While we provide a detailed and novel approach that combines both diet and gut microbiota, the sample size is limited, and we do not have longitudinal data. Hence, confirmation in larger, ideally population-based, rather than clinical cohorts is needed, ideally with longitudinal follow-up. Another potential limitation is our diet assessment tool, as it does not accurately determine gut microbiota accessible carbohydrates. Future dietary questionnaires should be optimized to capture relevant food-based microbiota modulators [5, 34]. Also, food diaries were collected 10–14 days before the stool sampling, but 3 to 4 days of dietary records are enough to be a representative [46]. Besides, further improvement of food tree configurations such as optimization of distance metrics used is desirable. Nevertheless, this study expands our knowledge of microbiota–diet association and provides new insight into the altered function of the gut microbiota in individuals with severe IBS symptoms, potentially as a consequence of interactions with dietary habits.

## Conclusions

In this study, we have demonstrated that individuals with severe IBS symptoms have a higher consumption of low-quality food products during their main meals and an enrichment of gut microbiota function towards a specific type of hydrogen metabolism associated with animal carbohydrate metabolism, detected using metagenomics. Those specific microbial metabolic pathways and metabolites should be targeted in future studies as markers for dietary/microbiota-targeted therapies for IBS. Our findings can potentially guide new interventional studies that aim to identify gut microbiome-based nutritional recommendations for the management of gastrointestinal symptoms.

## Supplementary Information

**Additional file 1: Figure S1.** Nutrients more abundant in the diets of healthy controls than in those of individuals with IBS. Absolute *Z*-scores between controls and individuals with IBS are shown. A positive *Z*-score indicates enrichment of the diet in the nutrient concerned and is shown in red for healthy controls and in blue for individuals with IBS. Significant *p*-values (uncorrected for multiple tests) are shown in a darker color.

**Additional file 2: Figure S2.** Comparison of nutrients between individuals with severe IBS symptoms and the other members of the study population (healthy, or individuals with mild or moderate IBS). Absolute *Z*-scores for comparisons between individuals with severe IBS symptoms and the other members of the study population are shown. Positive *Z*-scores, indicating nutrient depletion in individuals with severe IBS symptoms, are shown in blue. Significant *p*-values (uncorrected for multiple testing) are shown in a darker color.

**Additional file 3: Figure S3.** CAZotype as a function of the relative abundance of CAZy genes. CAZotypes 1 and 3 were notably enriched in glycosyl hydrolase (GH) 13 and carbohydrate-binding module (CBM) 26, both involved in starch metabolism, whereas CAZotype 2 was enriched in GH2 and CBM32. CAZotype 3 displayed a particular depletion of GH2 and GH20, which are known to be involved in mucin degradation.

**Additional file 4: Figure S4.** CAZotype and enterotype assignment, by individual.

**Additional file 5: Supplementary Table S1.** Food items studied and their associated hierarchical classification. **Table S2.** meat plan ratio and diet based PCoA components per individual. **Table S3.** Spearman correlation analysis between clinical and microbiome variable with co-inertia components.

## Data Availability

The datasets used and analyzed in this and the parent study [19] are available from 10.5878/ejpj-p674 and 10.5878/5rbz-ww62. Sequence data associated with this project have been deposited in EMBL under BioProject accession PRJEB34992. The source codes used in this study are available from GitHub (https://github.com/danone/zephyr.IBSFood).

## Abbreviations

CAZy: Carbohydrate-active enzymes
DMM: Dirichlet multinomial mixture
FODMAP: Fermentable oligosaccharide, disaccharide, monosaccharide and polyol
FSA-NPS: British Food Standards Agency Nutrient Profiling System
GI: Gastrointestinal
GOS: Galacto-oligosaccharide
IBS: Irritable bowel syndrome
IBS-SSS: IBS Severity Scoring System
JSD: Jensen Shannon divergence
MSPs: Metagenomics species pangenomes
PCoA: Principal Coordinate Analysis
