# Supplementary figures and images for: Diet and gut microbiome interactions of relevance for symptoms in irritable bowel syndrome
Source: Microbiome. 2021 Mar 26;9:74. doi: 10.1186/s40168-021-01018-9 (PMC8004395; doi:10.1186/s40168-021-01018-9)

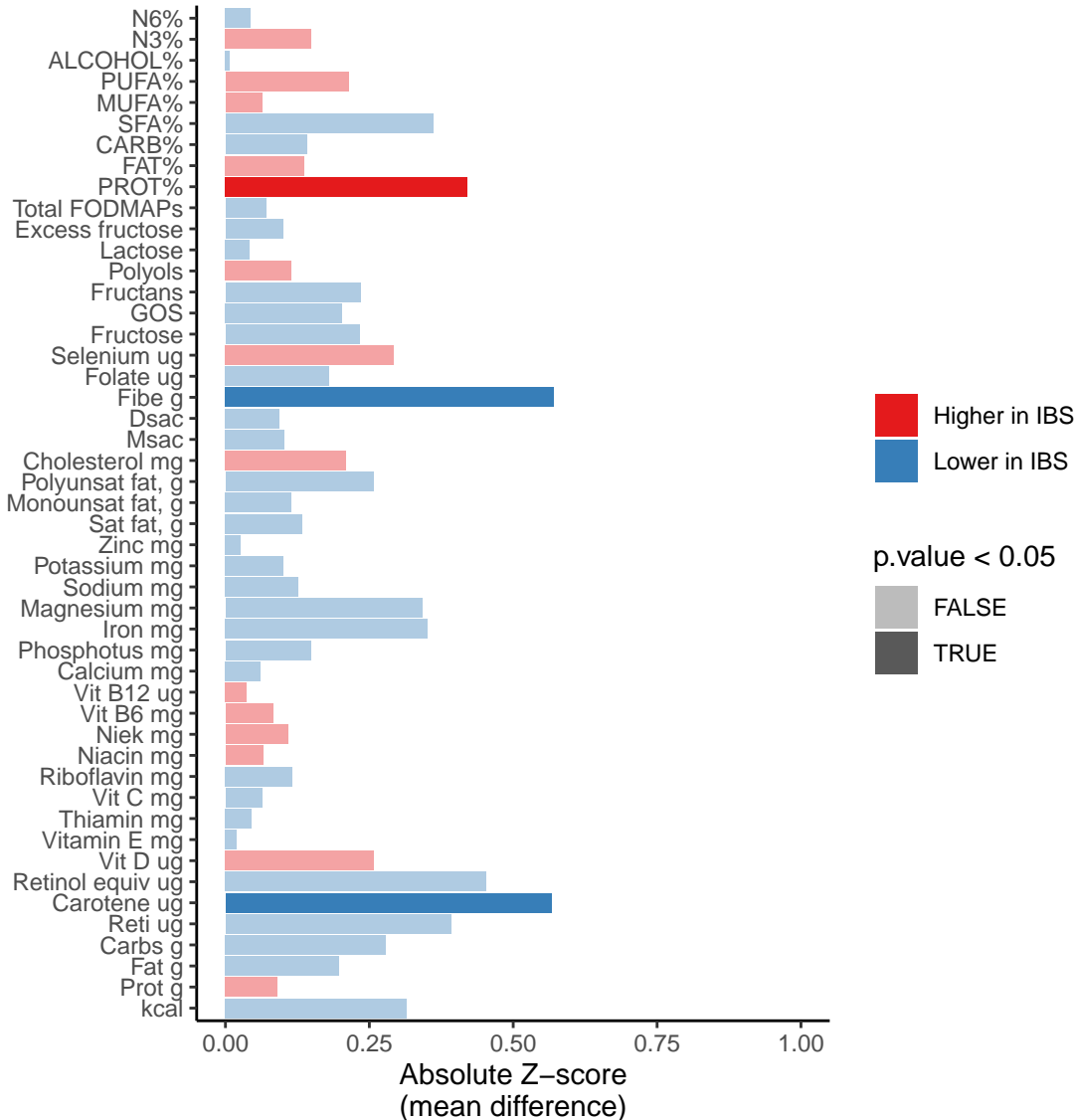

Supplement: Supplementary file 2 — Additional file 1: Figure S1. Nutrients more abundant in the diets of healthy controls than in those of individuals with IBS. Absolute Z-scores between controls and individuals with IBS are shown. A positive Z-score indicates enrichment of the diet in the nutrient concerned and is shown in red for healthy controls and in blue for individuals with IBS. Significant p-values (uncorrected for multiple tests) are shown in a darker color. [file 40168_2021_1018_MOESM2_ESM.pdf]

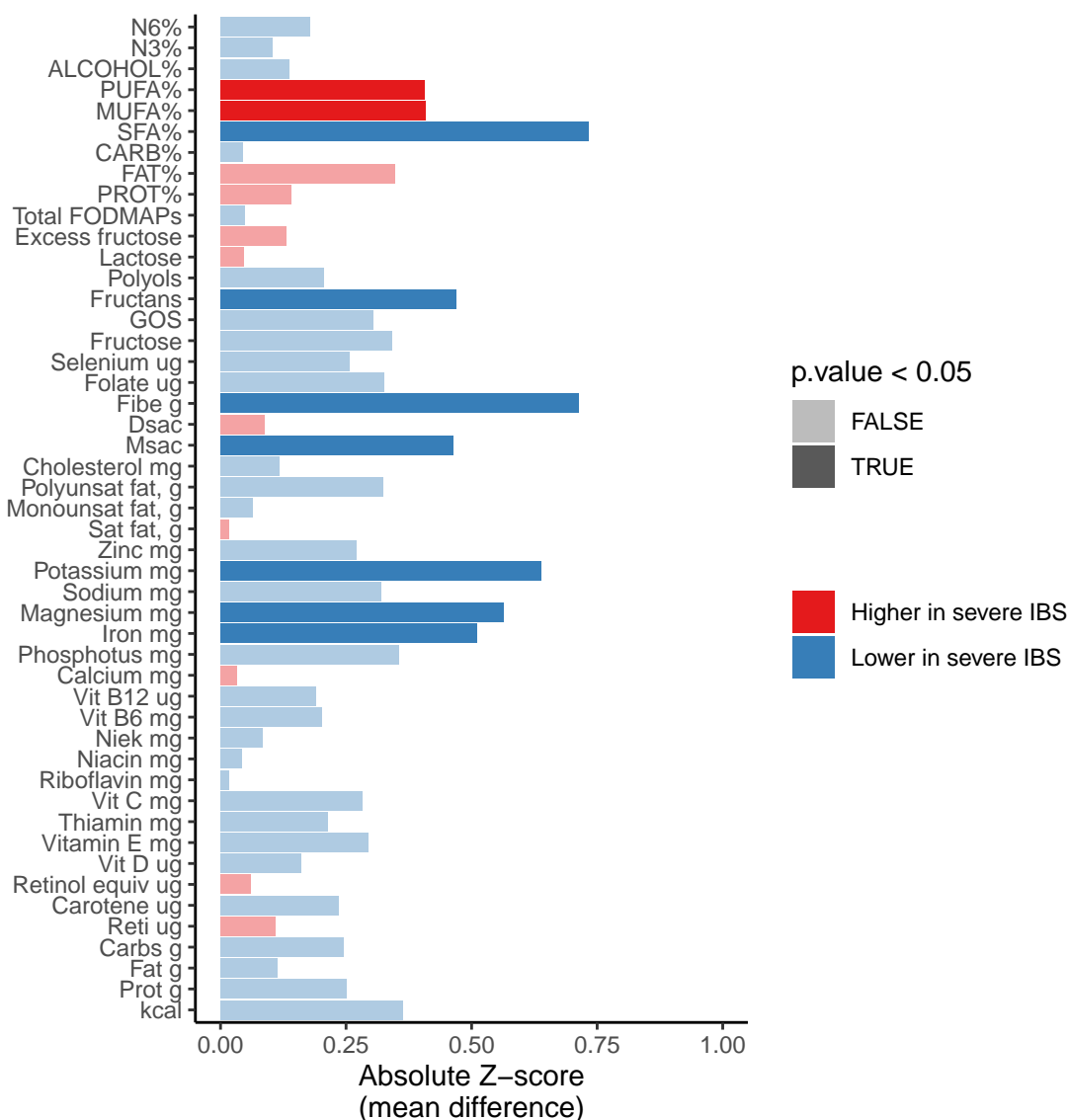

Supplement: Supplementary file 3 — Additional file 2: Figure S2. Comparison of nutrients between individuals with severe IBS symptoms and the other members of the study population (healthy, or individuals with mild or moderate IBS). Absolute Z-scores for comparisons between individuals with severe IBS symptoms and the other members of the study population are shown. Positive Z-scores, indicating nutrient depletion in individuals with severe IBS symptoms, are shown in blue. Significant p-values (uncorrected for multiple testing) are shown in a darker color. [file 40168_2021_1018_MOESM3_ESM.pdf]

Figure S3

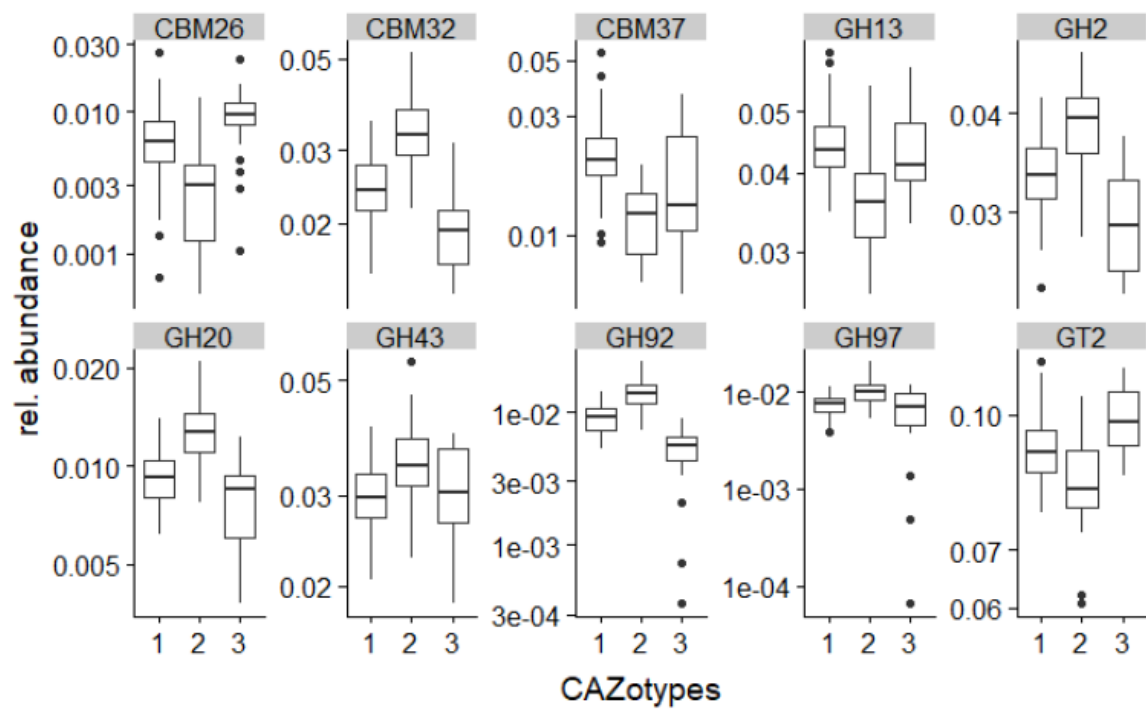

Supplement: Supplementary file 4 — Additional file 3: Figure S3. CAZotype as a function of the relative abundance of CAZy genes. CAZotypes 1 and 3 were notably enriched in glycosyl hydrolase (GH) 13 and carbohydrate-binding module (CBM) 26, both involved in starch metabolism, whereas CAZotype 2 was enriched in GH2 and CBM32. CAZotype 3 displayed a particular depletion of GH2 and GH20, which are known to be involved in mucin degradation. [file 40168_2021_1018_MOESM4_ESM.pdf]

Figure S4

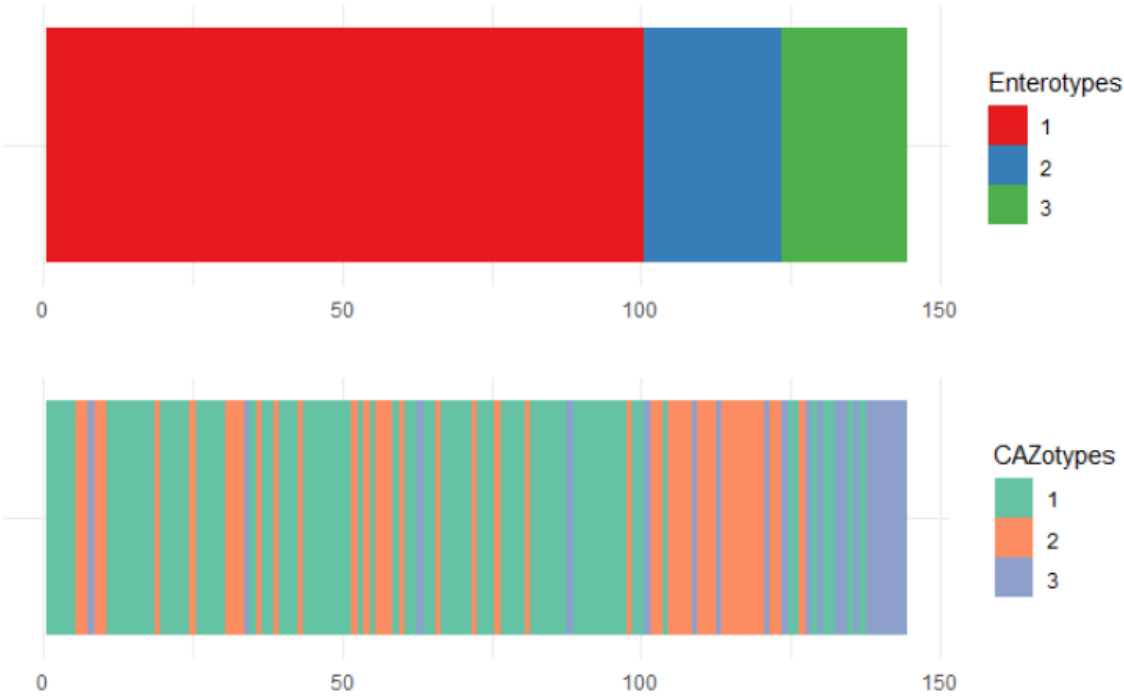

Supplement: Supplementary file 5 — Additional file 4: Figure S4. CAZotype and enterotype assignment, by individual. [file 40168_2021_1018_MOESM5_ESM.pdf]
